# Knockdown of *GmD53a* confers strigolactones mediated rhizobia interaction and promotes nodulation in soybean

**DOI:** 10.7717/peerj.12815

**Published:** 2022-01-20

**Authors:** Naveed Rehman, Fahim Ullah Khan, Muhammad Imran, Shahid Ali Rajput, Yiming Li, Ihteram Ullah, Rana waseem Akhtar, Muhammad Imran, Arwa Abdulkreem AL-Huqail, Ahmad El Askary, Amany Salah Khalifa, Muhammad Tehseen Azhar

**Affiliations:** 1 State Key Laboratory for Conservation and Utilization of Subtropical Agro-Bioresources, Guangdong Provincial Key Laboratory of Plant Molecular Breeding, South China Agricultural University, Guangzhou, China; 2 Department of Agriculture, Hazara University, Dodhial, Mansehra, Mansehra, Khyber Pakhtunkhwa, Pakistan; 3 Department of Agriculture, South China Agricultural University, Guangzhou, Guangdong, China; 4 Faculty of Veterinary and Animal Sciences, Muhammad Nawaz Shareef University of Agriculture, Multan, Punjab, Pakistan; 5 Department of Plant Breeding & Genetics, Gomal University, Dera Ismail Khan, Khyber Pakhtunkhwa, Pakistan; 6 Department of Biology, College of Science, Princess Nourah Bint Abdulrahman University, Riyadh, Saudi Arabia; 7 Department of Clinical Laboratory Sciences, College of Applied Medical Sciences, Taif University, Taif, Saudi Arabia; 8 Department of Clinical Pathology and Pharmaceutics, College of Pharmacy, Taif University, Taif, Saudi Arabia; 9 Institute of Molecular Biology and Biotechnology, Bahauddin Zakariya University, Multan, Punjab, Pakistan; 10 School of Agriculture Sciences, Zhengzhou University, Zhengzhou, China

**Keywords:** *GmD53a*, Nitrogen fixation, Rhizobia positive interaction, Symbiotic, Biotic factor, Sustainability, Food security, Signaling, Soybean nodulation, Strigolactone

## Abstract

**Background:**

Strigolactones (SLs) play a key role in modulating plant root growth, shoot branching, and plant-symbiont interaction. However, despite their significance, the components of SL biosynthesis and signaling in soybean and their role in soybean-rhizobia interaction is unknown.

**Methods:**

In this study, we identified and functionally characterized the *GmD53a* from soybean. The *GmD53a* ORFs were amplified from root cDNA using primers for *GmD53a* RNA interference. To induce transgenic hairy roots of soybean, electric shock was used to transform pB7WG1WG2 vectors containing *GmD53a* knockdown and *GUS* into *K599* strains of *Agrobacterium rhizogenes*. The hairy roots and nodules were collected and examined for root nodules ratio and RNA was extracted after 4 weeks of rhizobia inoculation.

**Results:**

A tissue-specific expression assay showed that *GmD53a* was differentially expressed in plant parts, predominantly in the stem and nodule. Furthermore, its expression was significantly up-regulated during rhizobia infection and varied with nodule formation. The *GmD53a*-knockdown chimerical plants were produced to further check its role in soybean nodulation in comparison with control *GUS*. In knockdown lines, the *GmD53a* (suppressor of strigolactone MAX2) has a higher number of nodules compared to control lines. Furthermore, the expression levels of several nodulation genes essential for initiation and formation of nodules were altered in *GmD53a*-knockdown lines.

**Conclusion:**

The results revealed that SL biosynthesis and signaling are not conserved but also have close interaction between SL and legume rhizobia.

## Introduction

Strigolactone (SLs), a new class of terpenoid lactones is generated from carotenoids, and it was identified as a component of root secretion for parasitic witchweed germination (Xie, Yoneyama & Yoneyama, 2010). SLs are required for the formation of symbiotic arbuscular-mycorrhizal fungi in plants which are effected by phosphorus deprivation (Akiyama, Matsuzaki & Hayashi, 2005; Bouwmeester et al., 2003; Waldie, McCulloch & Leyser, 2014), and has also been involved significantly in rhizobia-legume interaction (Foo et al., 2013; Liu et al., 2013; Liu et al., 2011). Rhizobia-legume interaction also activates defense responses and increases salt tolerance in soybean seedlings (Qu et al., 2016). In addition, under water deficit conditions, inoculation of rhizobia on soybean resulted in an increased number of nodules (Kibido et al., 2020).

The discovery of strigol supported the agricultural community in understanding and developing system to increase nitrogen fixation. Genetically, SLs are associated with numerous shoot mutants, such as *Ramosus* of pea (*Pisum sativum*) (Beveridge, Symons & Turnbull, 2000), More Axillary Growth (*MAX*) of *Arabidopsis thaliana* (Booker et al., 2005), Decreased Apical Dominance (*DAD*) of petunia (*Petunia hybrida*) (Simons et al., 2007) and Dwarf or High-Tillering Dwarf(*D/HTD*) of rice (*Oryza sativa*) (Arite et al., 2007; Lin et al., 2009). The SLs are found to be synthesized from carotenoid pathways employing a carotenoid biosynthesis inhibitor and carotenoid metabolism mutants (Matusova et al., 2005). In addition, rice mutants *d17* and *d10* in CCD7 and CCD8 were found to have defective mechanisms, and *ccd8/rms1* deficiency in SL synthesis was discovered in mutants of pea (Gomez-Roldan et al., 2008; Umehara et al., 2008). The roles of *CCD7* and *CCD8*, *MAX1*, and P450 cytochrome in the production of SLs have also been discovered. However, strong evidence is required to prove that SLs are made up of carotenoids (Crawford et al., 2010). Lin et al. (2009) also found the Fe-containing protein DWARF27 (D27) in the biosynthesis of SLs. SLs biosynthesis genes *D27, CCD7*, and *CCD8*; the three primary biosynthetic enzymes that complete the sequential processes and produce CL as a product (Alder et al., 2012). *D27* catalyzes the reversible isomerization of all-Trans-β-carotene at the C-9 position to create 9-cis-β -carotene. First *CCD7* utilizes all-Trans-β-carotene as a substrate and generated all-trans-β-10-carotenol, and then the *CCD7* cleaves the 9-cis-carotene and converts it to the 9-cis-apo-10-carotenol, which is consumed by *CCD8* to make CL (Schwartz, Qin & Loewen, 2004). A P450 mono-oxygenase, encoded by *Arabidopsis MAX1* and lotus *LBO* further converts carlactone into 5-deoxystrigol and other bioactive SLs (Abe et al., 2014; Breakspear et al., 2014). Furthermore, rice *D14* or petunia *DAD2*, α/β-fold hydrolase that can hydrolyze SLs and act as an SL receptor. In the presence of SLs, MAX2/D3 group of F-box proteins binds with D14 for the production of D14/Skp1–Cullin–F-Box (SCF) E3 ubiquitin ligase complex D14-SCFD3/MAX2 (De Saint Germain et al., 2016; Hamiaux et al., 2012; Stirnberg, Furner & Ottoline Leyser, 2007; Yao et al., 2016). Notably, MAX2 and SCF (Skp1–Cullin–F-Box) play a critical role in SL-triggered protein degradation (Zhao et al., 2014). D53 proteins can create D53–D14–SCFD3 protein complexes by interacting with D14 and D3 proteins. These allow the proteasome system to preferentially degrade the ubiquitin D53 protein, and activate the expression of downstream target genes that lead to the regulation of tillering in cereal crops. This means that tillering ability can be influenced by modulating *D53* expression, which can alter the SL signal transduction (Lv et al., 2019).

Although D53 protein belongs to the small group or family SMAX1-like (SMXL) and shares similarities with enzymes such as CIp ATPase enzymes, little is known about how D53 protein suppresses SLs signaling (Stanga et al., 2013). SLs are generated in roots and stems and transported upwards through the xylem to higher regions of the plant or extruded into the extracellular spaces (Kohlen et al., 2011). An ATP-binding cassette (ABC) transporter Pleiotropic Drug Resistance1 (PDR1) was identified as a SL exporter (Kretzschmar et al., 2012). Despite significant advances in understanding SL production and signaling, many SL-related events, such as complicated cross-talks or interactions between SLs and other hormones remain unknown.

Recently, it has been found that SL biosynthesis and signal transduction may play a significant role in soybean nodulation (Ahmad et al., 2020; Bennett & Leyser, 2014; Bennett et al., 2016; Rehman et al., 2018). Because the *D53* gene is important for tillering regulation, we used RNAi techniques to knock down the homologous soybean gene of D53 (*GmD53*a), and examined the physicochemical properties and structure of GmD53 protein using bioinformatics tools. Furthermore, the role of GmD53a protein in soybean root nodulation was demonstrated in this study.

## Materials and Methods

### Plant growth conditions

Seeds of soybean were surface sterilized with sodium hypochlorite (NaClo) and hydrochloric acid (HCL) and grown in three-gallon pots containing vermiculite soils at National Key Laboratories, Huazhong Agricultural University, Wuhan. The seedlings were grown at 26/20 °C (day/night) temperature, photoperiod of 14/10 h, 800 µmol m^−2^ s^−1^ light intensity and 60% humidity was maintained in growth chamber. Seeds, stems, roots, flowers, leaves, of and pods from soybean cultivar (Tianlong no. 1) were harvested at different growth phases. After treatments, all selected tissues were sensibly removed and instantly put in liquid nitrogen and then stored at −80 °C. Then, RNA was extracted and later on cDNA was synthesized according the protocol of supplier.

### Construction of vector

The *GmD53a* (Glyma.11G230700.1) ORFs were amplified from root cDNA by using primers for *GmD53a* RNA interference (Table S1). Following that, RNA was isolated with TRIzole reagent (Invitrogen) or RNA kit (Biotech, Beijing) from soybean roots, and 10 g of RNA was taken in the cDNA synthesis utilizing the first-strand synthesis technique (Invitrogen) and cDNA was used as a template for the amplification of *GmD53a* as previously described (Ahmad et al., 2020). The amplified fragments were used for directionally cloning in pDONR221 vector *via* BP clonase and then recombined into the pB7GWIWG2 destination vector *via* LR clonase (Life Technologies, Rockville, MD, USA) after ORFs were cloned into T-easy vector (Promega, Madison, WI, USA) and sequenced. For comparison, the GUS gene was also recombined into both vectors. Then the constructs were transformed into *A. rhizogenes K599* by electroporation and were utilized to induce hairy roots.

### Induction of transgenic hairy root and nodulation assay GmD53a

To induce transgenic hairy roots of soybean, electric shock was used to transform pB7WG1WG2 vectors containing *GmD53a* knockdown and *GUS* into *K599* strains of *Agrobacterium rhizogenes*. *A. rhizogenes* strain K599 harboring pB7GWIWGII-Gm53a, cDNA fragments for knockdown constructs, or *GUS* were grown on LB-agar medium at 28 °C with spectinomycin and streptomycin as selection markers. The overnight *Agrobacterium* cultures were used for transformation of soybean cultivar. Seven days old seedlings of soybean were wounded at the hypocotyls region before being incubated in high humidity for 24 h carrying *A. rhizogenes* constructs (Ahmad et al., 2020). Hairy roots were emerged from the wounding sites after one week of infection, whereas non-transgenic roots were removed after one week. About 1 week after hairy root emergence when transgenic hairy roots were about to support the plants, the chimeric soybean plants were examined for transgene expression in hairy roots, and the main of non-transgenic roots are removed, before being inoculated with rhizobia strain USDA110 cultured in the YMA at 28 °C. About 25 mL rhizobia bacteria solution (OD 600 nm) was applied to each plant pot. The hairy roots and nodules were examined and collected for root nodules ratio, and RNA was extracted after 4 weeks of inoculation. Three independent experiments of transformation with 10 individual transgenic lines under equal treatments and growth conditions were carried out for each vector including *GUS* as a control. We determined the numbers of nodules from hairy roots per grams for normalization.

## Rhizobia infection

For the rhizobia infection experiment, seedlings of about 14 days old were inoculated with “USDA-110” (O.D_600_ = 0.08–0.1) strain of *Bradyrhizobium japonicum* in the YMA medium. Roots of infected plants were collected at 0, 6, 12, 24, 36, and 48 h after inoculation. These frozen tissues were used the extraction of RNA to synthesize cDNA.

## QRT-PCR analysis of gene expression

Total RNA was isolated from several tissues *i.e*., seeds, leaf, stem, flower, root, and nodules), or transgenic hairy roots by using TRIzol (Invitrogen, Carlsbad, CA, USA) or RNA kit (Biotech, Beijing) according to instructions from the manufacturer. About 10 g of total RNA was digested with RNase-free DNaseI (Promega, Madison, WI, USA) to remove any genomic DNA contamination for each sample. A NanoDrop ND-2000 UV spectrophotometer (Thermo Scientific, Waltham, MA, USA) was used to measure the concentration of RNA. The first strand cDNA was prepared from 10 µg total RNA using the Superscript III first strand synthesis system (Invitrogen). All of cDNA samples were diluted with sterile water according to the requirements for qRT-PCR analysis where gene-specific primers were used (Table S1). The reaction mixture of 20 µL containing 2.5 µL SYBR Master Mix (Applied Biosystems), 1 µL primer mix (0.4 µL from each primer, 0.2 µL ddH2O), and 2 µL of 100 ng cDNA were put in 96-well plates (iQ5 Real Time PCR System; Bio-Rad) for all of selected tissues. The transcript levels were normalized by using *GmACTIN1* as previously described (Ahmad et al., 2020).

## Bioinformatics analysis

Protein sequences of SLs signaling genes reported from monocots and dicots were obtained from NCBI or phytozome database (phytozome.jgi.doe.gov/) for phylogenetic analysis and identification of SLs signaling genes. The phylogenetic tree was constructed by MEGA6, and Neighbor-joining was performed with 1,000 bootstraps (Tamura et al., 2013). Bioinformatics analyses on protein sequences of SL pathway genes were obtained from NCBI (https://www.ncbi.nlm.nih.gov/) and phytozome (phytozome.jgi.doe.gov/). The sequence similarities and identities were determined from Blast2 (http://pga2.mgh.harvard.edu:8080/rtpcr/blast/wblast2_cs.html). The RNA Seq-Atlas data from nine soybean tissues were extracted from public databases (http://soybase.org/soyseq/) to acquire the tissue-specific expression and stages of nodule development.

## Statistical analysis

The data were recorded from three independent experiments for each trait, and Student’s *t*-test was applied for analysis using Statistix 8.1 software. The confidence limit 95% represents the significant between two-tailed data (Rehman et al., 2018).

**Figure 1:**
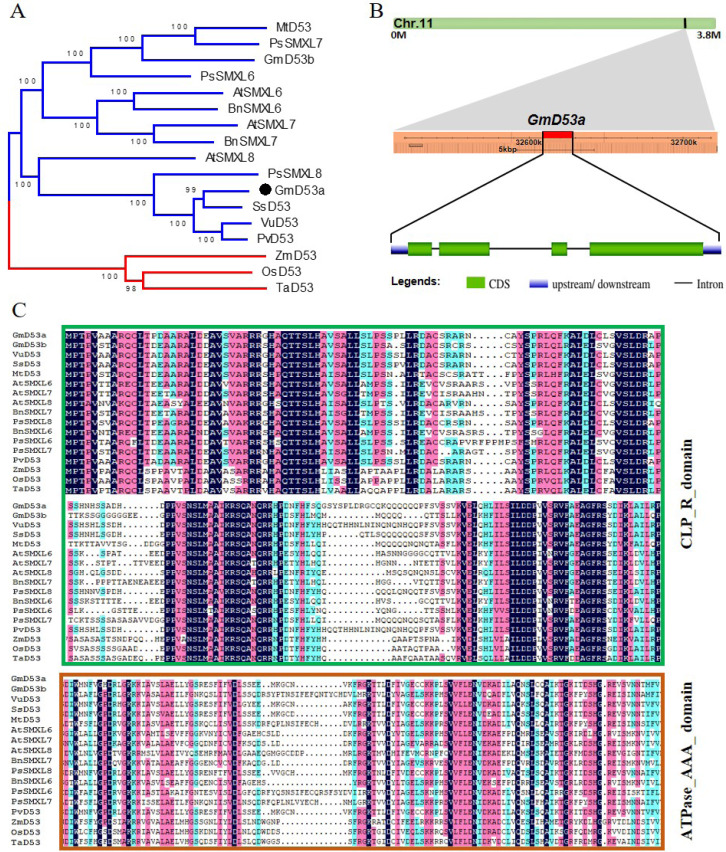
Distinct regions of GmD53a exhibit sequence conservation. (A) Phylogenetic tree of GmD53a from dicotyledonous and monocotyledonous plants. (B) Genomic position and gene structure of GmD53a. (C) The CLP-R and ATPase-AAA conserved domain regions with respect to other members.

## Results

### Identification of SL biosynthetic genes from soybean genome

The homologs of other organisms with 1 × 10^−3^ permissive E cut-off values were discovered in tBLASTp searches to assess the evolutionary relationship of GmD53 with other plant species. A neighbor-joining phylogenetic tree was generated by using the D53 proteins from 12 dicotyledons and three monocotyledons by using MEGA6.0 software to compare the evolutionary relationships between GmD53a and other plant D53 proteins (Fig. 1A). The phylogenetic analysis revealed that all D53 proteins were divided into two main groups, *i.e.,* dicotyledonous D53 proteins and monocotyledonous D53 proteins, indicating that dicotyledon D53 proteins were highly homologous. This group included all of dicotyledon D53 proteins which were separated into three sub-groups. One sub-group had D53 proteins from Orchidaceae, Palmae, and Musaceae, while other sub-groups were composed of D53 proteins from Gramineae. Thus, D53 proteins from Gramineous are closely related, and ScD53 protein was closely related to sorghum (*Sorghum bicolor*) D53 protein followed by maize (*Zea mays*) and millet (*Panicum miliaceum*). Furthermore, homology analysis by using Blastp program in NCBI showed that ScD53 had 93%, 82%, 80%, and 69% identity with D53 proteins of sorghum, maize, foxtail (*Setaria italic*), and rice (*Oryza sativa*), respectively. Besides, GmD53a was located on chromosome number 11, and exon/intron distribution analysis showed that GmD53a contains four exons and three introns with varying lengths (Fig. 1B). Further analysis of GmD53a protein sequence alignment revealed that all the 17 members were delimited by CLP-R and ATPase-AAA domain (Fig. 1C), suggesting that GmD53a is evolutionarily conserved in plant species.

### Tissue-specific and altered expression in nodule stages of *GmD53a*

**Figure 2:**
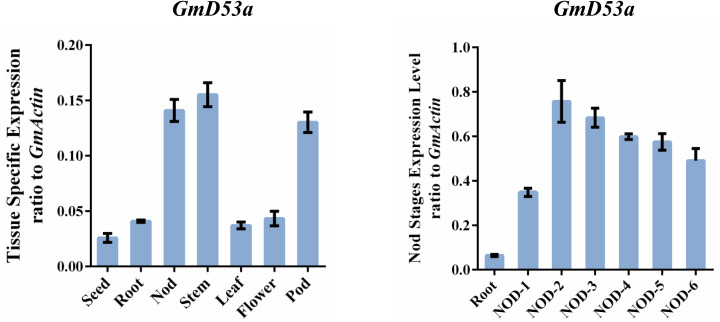
Tissue-specific expression patterns of *GmD53a*. (A) Relative expression of GmD53a to *GmACTIN* was measured with samples from soybean cultivar “Tianlong 1” at different development stages by using qRT-PCR. (B) Relative expression of GmD53a gene throughout nodule development and senescence using qRT-PCR. Expression levels of *GmD53a* were examined by qPCR and normalized to the average expression level of *GmACTIN*. Data are expressed as means ± s.d. from three independent experiments with biological replicates.

The expression patterns of the strigolactone repressor *GmD53a* gene were assessed in various tissues and organs. We performed a BLAST search of the rice *D53* gene in Phytozome and obtained two close homologs *GmD53a* and *GmD53b*. We have compared gene expression patterns to public databases (http://soybase.org/soyseq/) *GmD53a* (Figs. 2: S1) to understand the role of these genes. Data on the expression levels of genes implicated in strigolactone signaling in nine different plant tissues (pod, root hair, root, nodule, stem, leaf, seed, shoot apical meristem (sam), stem, and flower) were studied. In soybean plants, we explored how the *GmD53a* gene was expressed in different tissues and organs. *GmD53a* was most abundant in the stem, pods, nodules, and leaves, while *GmD53b* was abundant in the stem, Shoot Apical Meristem (SAM), and leaves (Fig. S1). However, low expression was observed in the remaining tissues (Figs. 2, S1). *GmD53a* was more abundant in most of tissues and organs, particularly roots and nodules, than *GmD53b* (Fig. S1).

In addition, we also examined the expression of *GmD53a* gene, which is involved in SL signaling, throughout nodule development and senescence. We evaluated the RNA-Seq data from soybean nodules at various phases of development and validated it with qRT-PCR. The results revealed that *GmD53a* was differently expressed in nodules at various stages of development till nodule senescence (Fig. S2). The data were validated through qRT-PCR which was consistent with the RNA-Seq results. Interestingly, the transcript levels of *GmD53a* increased significantly during growth stages of nodule formation, and it was maximum at stage 2, and then declined afterward (Fig. 2).

### Effect of *GmD53a-knockdown* on soybean nodulation

**Figure 3:**
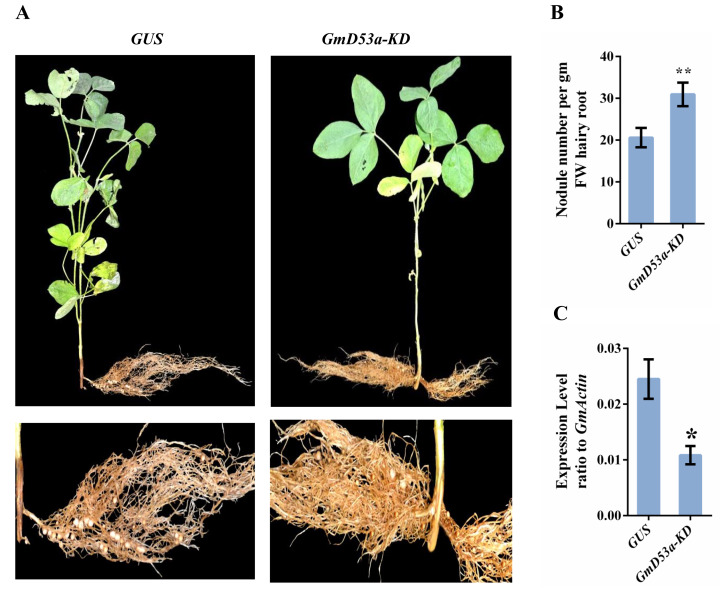
Effects of *GmD53a-knockdown* on soybean nodulation. Chimerical soybean plants were generated by transformation with K599 harboring, *GmD53a-knockdown*, or *GUS* vector. Plants with wild-type shoots and transgenic hairy roots were inoculated with *Bradyrhizobium japonicum* strain USDA110. Nodule numbers from the hairy roots were counted and the roots were sampled for gene expression analysis at 28 days post inoculation. (A) Chimerical soybean plants with wild-type shoots but transgenic hairy roots. *GmD53a-KD* plants developed less nodules as compared with *GUS* control. (B) Hairy root fresh weight (g) and nodule numbers ratio in *GmD53a-KD* and *GUS* control lines. (C) qRT-PCR confirmation of *GmD53a-KD* in transgenic hairy roots as compared to *GUS* control. Gene expression was determined by qRT-PCR with *GmACTIN* as an internal control. Data are expressed as means ± s.d. from at least 3 independent experiments with biological replicates. Differences were analyzed, *p < 0.05; **p < 0.01 in student’s *t*-test.

The knockdown chimerical soybean plants known as *GmD53a-KD* were developed to investigate the function of SLs *Dwarf* gene *GmD53a* in soybean nodulation, (Fig. 3A). The plants were infected with *GmD53a* containing RNAi vector and were collected after four weeks of rhizobium inoculation. The ratio of hairy roots to nodules was examined, and it was found that *GmD53a* had substantially more nodules per gram of fresh-weight hairy roots than *GUS* control (Fig. 3B). *GmD53a-KD* plants produced 60% more nodules than *GUS* control plants (*P* < 0.01). This change in number of nodules in *GmD53a-KD* plants was seen in three separate trials. However, the total size of mature root system did not differ among knockdown and *GUS* control plants, unlike the number of nodules on mature roots. The qRT-PCR was used to analyze the genetic background of *GmD53a-KD*, which revealed the considerable repression of *GmD53a-KD* plants (*P* < 0.05) (Fig. 3C). The number of nodules on *GmD53a-KD* plants differed significantly from the control (*GUS*) plants (Fig. 3). As a result, *GmD53a* in SL signaling pathway acted as an antagonist in soybean nodulation.

### Response to rhizobia infection of *GmD53a-KD* hairy roots

**Figure 4:**
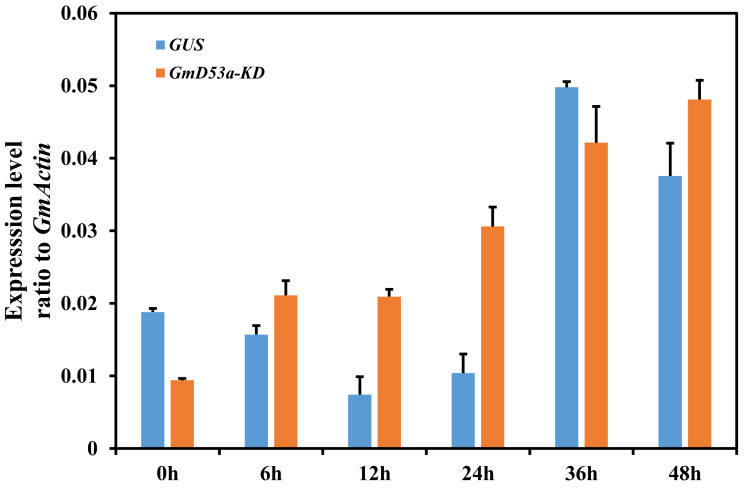
Effect of Rhizobia inoculation on *GmD53a-KD* transgenic lines. Chimerical soybean plants were generated by transformation with *K599* harboring, *GmD53a-knockdown*, or *GUS* vector. Plants with wild-type shoots and transgenic hairy roots were inoculated with *Bradyrhizobium japonicum* strain USDA110. Roots were collected at different time point after inoculation at 0 h, 6 h, 12 h, 24 h, 36 h and 48 h. Gene expression was determined by qRT-PCR with *GmACTIN* as an internal control. Data are expressed as means ± s.d. from at least 3 independent experiments with biological replicates. Differences were analyzed, *p < 0.05; **p < 0.01 in student’s *t*-test.

Apart from known activities in plant-mycorrhizal interactions, SLs have been reported to alter legume nodulation (Foo & Davies, 2011). The *GmD53a* was expressed after infection with *B. japonicum* (USDA110). To further explore how SL operates in soybean nodulation and expression of SL suppressor *GmD53a* in response to *B. japonica* infection, the *GmD53a-KD* and *GUS* control chimerical hairy roots were infected with rhizobia strain USDA110, and expression was assessed at various intervals after infection (Fig. 4). The results showed that *GmD53a* expression in hairy roots of *GUS* controls was reduced at 6 h and continued to decline significantly at 12 and 24 h (Fig. 4). Similarly, *GmD53a* expression was maximum at 36 h and it was declined at 48 h (Fig. 4). The expression level of *GmD53a* was increased considerably at every time point and peaked at 48 h as compared to *GUS* control (Fig. 4).

### Effect of *GmD53a*-knockdown on nodulation signaling pathway genes

**Figure 5:**
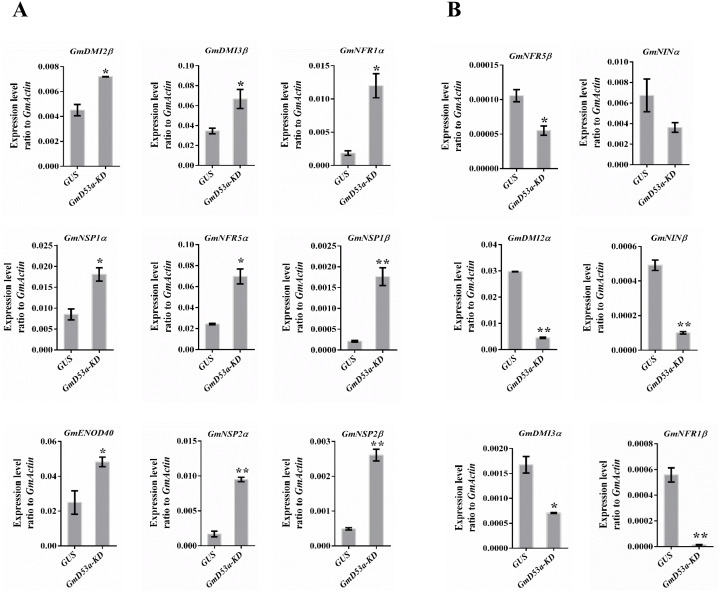
*GmD53a-KD* altered expression levels of nodulation genes. Expression level of nodulation genes in *GmD53a-KD* and *GUS* transgenic hairy roots as control. Gene expression was determined by qRT-PCR with *GmACTIN* as an internal control. Data are expressed as means ± s.d. from at least 3 independent experiments with biological replicates. Differences were analyzed, *p < 0.05; **p < 0.01 in student’s *t*-test.

Strigolactones are essential signaling molecules in plant-arbuscular mycorrhizal fungi interactions (Akiyama, Matsuzaki & Hayashi, 2005). Additionally, strigolactone influenced the nodulation in *Glycine max*, *Medicago sativa*, *Pisum sativum*, and *Lotus japonicas* (Ahmad et al., 2020; Foo et al., 2013; Liu et al., 2013; Soto et al., 2010; Rehman et al., 2018). Several genes are involved in symbiotic infection and nodulation was analyzed for expression by using qRT-PCR to see how knockdown of GmD53a exerts the effects on nodulation. Surprisingly, *GmD53a* knockdown was shown to dramatically up-regulated all of identified nodulation genes (Fig. 5). Among them, the cathway genes; does not make infection (*DMI2α*, GLYMA12G28860) were down-regulated while the expression of *DMI2β* (GLYMA16G00500) was found to be higher. A similar expression was recorded for *DMI3α* (GLYMA15G35070) and *DMI3β* (GLYMA08G24361) (Fig. 5). Nod Factor Receptor 1α (*NFR1α*; GLYMA02G43860) expression was considerably up-regulated in Gm53a-KD (P0.05), whereas *NFR1β* (GLYMA14G05060) expression was significantly down-regulated in *GmD53a-KD*. The expression of Nod Factor Receptor 5α (*NFR5α*; GLYMA01G38560) was considerably increased (*P* < 0.01) in *Gm53a-KD* as compared to control *GUS*, while the expression of Nod Factor Receptor 5β (GLYMA11G06740) was dramatically decreased (*P* < 0.01) in *Gm53a-KD* (Fig. 5). In *GmD53a-KD* lines against the *GUS*, the Nodule Inception α (*NINα*, GLYMA04G00210) was marginally lowered, while the (*NINβ*, GLYMA02G48080) was down-regulated. Nodulation Signaling Pathway 1 (*NSP1α*, GLYMA16G01020; *NSP1β*, GLYMA07G04430), and Nodulation Signaling Pathway 2 genes (*NSP2α*, GLYMA06G11610; *NSP2β* GLYMA04G43090) were significantly up-regulated (*P* < 0.01) in *Gm53a-KD* compared to *GUS* control (Fig. 5). The Early Nodulation 40 (*ENOD40*, Glyma01g03470) was up-regulated in *GmD53a-KD* gene compared to *GUS* control. Most of the nodulation pathway genes were significantly up-regulated in *GmD53a-KD* hairy root lines, which were in line with the result of rhizobia infection which might explain why *GmD53a-KD* transgenic hairy roots had more nodules than the *GUS* control (Fig. 5).

## Discussion

Soybeans are one of the important economic crops, and popularity is increasingly due to their role in nitrogen-fixing capabilities in animal feeds, the food sector, and sustainable agriculture. Understanding the role of SLs in soybeans is critical for the development of new accessions of soybean because the cited gene plays crucial roles in controlling the plant architecture, shoot branching, root growth, plant-mycorhization, and legume-rhizobium interactions. While the literature on studies on SLs in soybeans is scanty because these genes have not been investigated thoroughly by many researchers. Therefore, this research work was planned to investigate the role of SL signaling genes in the development, nodulation and architecture of soybean.

Unlike diploid model legumes such as Medicago and Lotus, which have only one copy of homolog of the SL biosynthesis gene, while the tetraploid soybean genome has many copies of each *MAX* or *Dwarf* gene. Orthologs from pea, *Arabidopsis*, and rice are similar to each other. SLs are typically synthesized in roots and stems and transported upward to shoots and leaves in other plants (Kohlen et al., 2011; Yoneyama et al., 2008). SLs biosynthesis genes have higher expression in roots and stems, but SL signaling genes can be expressed anywhere in plants (Kohlen et al., 2011; Yoneyama et al., 2008). Indeed, the expression of *GmD53a* in soybean confirms the conserved SL signaling components. In addition, rhizobia infections have significantly altered the expression of SL suppressor *GmD53a*. According to another study on pea, SLs play a key role in the development of infection threads after rhizobia infection by influencing early nodulation gene expression (McAdam et al., 2017).

SLs are linked to legume nodulation, in addition to their physiological effects on root growth, shoot branching, and mycorrhizal branching (Foo, 2013; Foo & Davies, 2011; Foo et al., 2013). The pea SLs-deficient mutant *rms1/CCD8* produces 40% fewer nodules than the wild type, although synthetic SLs analog GR24 partially reversed the phenotype (Foo & Davies, 2011). SLs were reduced by 80% in *Lotus japonicus LjCCD7*-silenced plants, and nodules were reduced by 20% when compared to control plants (Liu et al., 2013; Liu et al., 2011). In *GmD53a-KD* soybean roots, the structural grounds for nodule initiation, development, and rhizobia interaction were significantly changed. As previously stated, root hair development and immediate responses of root hairs to rhizobial Nod factors are crucial for nodulation (Oldroyd, 2013). We also found that knocking down of *GmD53a*, a SL suppressor, boosted soybean nodulation. *GmD53a-KD* hairy roots had a 60% (*P* < 0.01) increase in nodule quantity compared to *GUS*, implying that *GmD53a-KD* influenced nodulation in chimerical transgenic hairy roots compared to control. The gene expression analysis revealed that *GmD53a* knockdown hairy roots also altered the expression of several key genes which are involved in nodulation. Similar to *CCD7* and *CCD8* in pea and *Medicago* (Liu et al., 2013; Liu et al., 2011; Van Zeijl et al., 2015), over-expression of *GmMAX1a, 2a, 3a*, and *4a* were also involved in nodulation initiation and development as evidenced by genetic and molecular data (Ahmad et al., 2020); ur (Rehman et al., 2018). On the other hand, soybean *GmD53a-KD* had other opposite effects on nodulation in soybean. It was previously established that increase in the expression of *D10* in *d53* mutant explains a feedback control of the SL pathway (Zhou et al., 2013). The same researchers have also reported that that in *d53* mutant, the expression of an axillary bud inhibitor FINE CULM 1 (*FC1*), which is an orthologue of TEOSINTE BRANCHED1 (*TB1*) and *Arabidopsis* BRANCHED 1 (BRC1) was down-regulated, implying that D53 is involved in SL biosynthesis or signaling (Zhou et al., 2013). Likewise, the accumulation of SLs in the roots of *d53* mutant was significantly higher than compared to WT (Zhou et al., 2013). The increase in number of nodules in *GmD53a-KD* describes that SLs level might be higher in roots of *GmD53a-KD* compared to *GUS* control.

In particular, the *d53* mutant plants had more number of tillers than the wild type (Zhou et al., 2013). The expression level in transgenics was linked with severity of tillering phenotype. In the same way, over-expression of *D53* gene resulted in a slight increase in tillering compared to control plants (Zhou et al., 2013). These findings strongly suggest that D53 protein functions as a suppressor of the SL-mediated branching inhibition pathway, and the more tillering phenotype of *d53* mutant was generated by a gain of function mutation in d53. RNA interference (RNAi) was used to develop *D53* knockdown transgenic plants; as expected, the number of tillers was reduced (Zhou et al., 2013). These findings support the hypothesis that *d53* mutation increases the *D53* activity in repressing SL signaling (Zhou et al., 2013). Notably, a decreased expression of *D53* significantly reduced the tiller number of *d3* and *d14* mutant lines, respectively, indicating that the reduction of *D53* expression could partially rescue the signaling defects of *d3* and *d14* and the result is consistent with its negative role in SL signaling (Jiang et al., 2013). It also validated the increased nodule number in *GmD53a-KD* lines compared to *GUS* control.

Downstream factors such as nodulin genes, and *ENOD40* are connected to *NFR1α* genes which are involved in Nod factor perception and involved in Nod factor signal transductions, namely, *DMI2α* and *DMI3β*, *NINα*, and *NSP2β* (Oldroyd, Engstrom & Long, 2001; Oldroyd, 2013). Our findings showed that *GmD53a* knockdown affects the nodulation signaling pathway.

*MtD27* expression in nodulation is influenced by several symbiotic signaling pathways, including *MtDMI1*, *MtDMI2*, and *MtDMI3/MtCCaMK*, in addition to *NSP1* and *NSP2* (van Zeijl et al., 2015). Nodulation, which is dependent on SL signaling, was disrupted in the *rms1/CCD8* pea SLs-deficient mutant (Foo & Davies, 2011). In *Lotus japonicas LjCCD7*-silenced plants, nodulation abnormalities were also found as compared to control plants (Liu et al., 2013; Liu et al., 2011). *GmD53a-KD* may affect nodulation in soybean by influencing nodule initiation genes (Fig. 5).

## Conclusion

In this study, the soybean genome was exploited to find closely related homologs of *SMXL6, 7, 8* from *Arabidopsis*, and *D53* from rice to further figure out whether and how soybean SL signaling biosynthesis is involved in controlling the various growth and developmental phases in the soybean plant. The results showed that *GmD53a* is involved in the nodulation of soybean. This study not only demonstrated that SL signaling is conserved across the reported crop plants but also revealed that how *GmD53a* controls the developmental phases and legume-rhizobia interaction. However, further investigating the roles of these signal components may provide potential targets for directional improvement of crop traits, and further research that focuses on these aspects is highly desired.

## Supplemental Information

10.7717/peerj.12815/supp-1 Supplemental Information 1 Primers used in this study

10.7717/peerj.12815/supp-2 Supplemental Information 2 Supplemental Figures

10.7717/peerj.12815/supp-3 Supplemental Information 3 Raw Data

## Additional Information and Declarations

### Data Availability

The following information was supplied regarding data availability: The raw data is available in the Supplemental File.
