# Supplementary material for: Knockdown of GmD53a confers strigolactones mediated rhizobia interaction and promotes nodulation in soybean
Source: PeerJ. 2022 Jan 20;10:e12815. doi: 10.7717/peerj.12815 (PMC8784017; doi:10.7717/peerj.12815)
Supplement: Supplemental Information 1 [file peerj-10-12815-s001.docx]

**TABLE S1. Primers used in this study**

| **RiD53**  (Glyma11G230700) | F:GGGGACAAGTTTgtacaaaaaagcaggctTCGTGAATACATCAATCTCAACTCCCA  R:GGGGACCACTTTGTACAAGaaagctgggtACATCTTGCCAGCATACCTCCT |
| --- | --- |
| **qRT-GmD53**  **(**Glyma11G230700**)** | F: TTTGTGAGCGATGAAGAAGGT  R:TTTGTGAGCGATGAAGAAGGT |
| **qRT-GmNFR1α**  (GLYMA02G43860) | F: ATTCACGAGCACACTGTGCCT  R: CCAAAATCTGCAACCTTTCC |
| **qRT-GmNFR1β**  **(**GLYMA14G05060) | F: CGATGGTGAGTTTCTGGGGCA  R: ATCAAGGCGAGCCTCGTTGG |
| **qRT-GmNFR5α**  (GLYMA01G38560) | F: TTCCCTTTCTTCCTCTCCAC  R: GCATGAAAAGTTTGTTCTATTGTC |
| **qRT-GmNFR5β**  **(**GLYMA11G06740) | F: GTGTGGAAGCCCGGTGACAA  R: CCGCCTTTTCTTCCGTCCGA |
| **qRT-GmDMI2α**  (GLYMA01G02451 ) | F: GTCCTCAGTGGCCTTGACATT  R: ACACCCTTTTGCCTGCTTTG |
| **qRT-GmDMI2β**  **(**GLYMA09G33510) | F: ACCATGCAGAGGCAATGTGG  R: GAGGGCAGCACCCTTTTCTC |
| **qRT-GmDMI3α**  (GLYMA15G35070) | F: AGTGTTTGGAGCACCGCAATC  R: TCAAACAAGTCAAATATACGTGGTG |
| **qRT-GmDMI3β**  **(**GLYMA08G24361) | F: GGAGAGAAGAGCACAGCAGC  R: ACAGCTCCAACACAAGGTGC |
| **qRT-GmNSP1α**  (GLYMA16G01020) | F: CAACACTTATCTTCTTCTCCAACT  R: GGAAGCATTTGCTATGTTGTTAGG |
| **qRT-GmNSP1β**  **(**GLYMA07G04430) | F: CCGTGGTCACTGCTTCTTCC  R: CCCTTTGGGCTCCATGTTCC |
| **qRT-GmNSP2α**  **(**GLYMA06G11610) | F: GAGGAGGAAGTGGCCTCCAT  R: TGATTCGCGAAGCTCATCGG |
| **qRT-GmNSP2β**  (GLYMA04G43090) | F: AATCATTGCCAAGCGAAGCT  R: AGTCCAAAGCGAGGCAGAGA |
| **qRT-GmEnod40**  (Glyma01g03470.1) | F: GAAAGGGGTGTGAGAGGAGAG  R: CGCCACTCAAGAAAGAATGTT |
| **qRT-GmNINα**  (GLYMA04G00210) | F: TAACATGCGATGCTGATCTTG  R: TGATTTAGAGGCGAAGCTTGA |
