# Supplementary material for: Knockdown of GmD53a confers strigolactones mediated rhizobia interaction and promotes nodulation in soybean
Source: PeerJ. 2022 Jan 20;10:e12815. doi: 10.7717/peerj.12815 (PMC8784017; doi:10.7717/peerj.12815)
Supplement: Supplemental Information 2 [file peerj-10-12815-s002.docx]

**GmD53a(GLYMA11G35410)**

**GmD53b(GLYMA18G06990)**


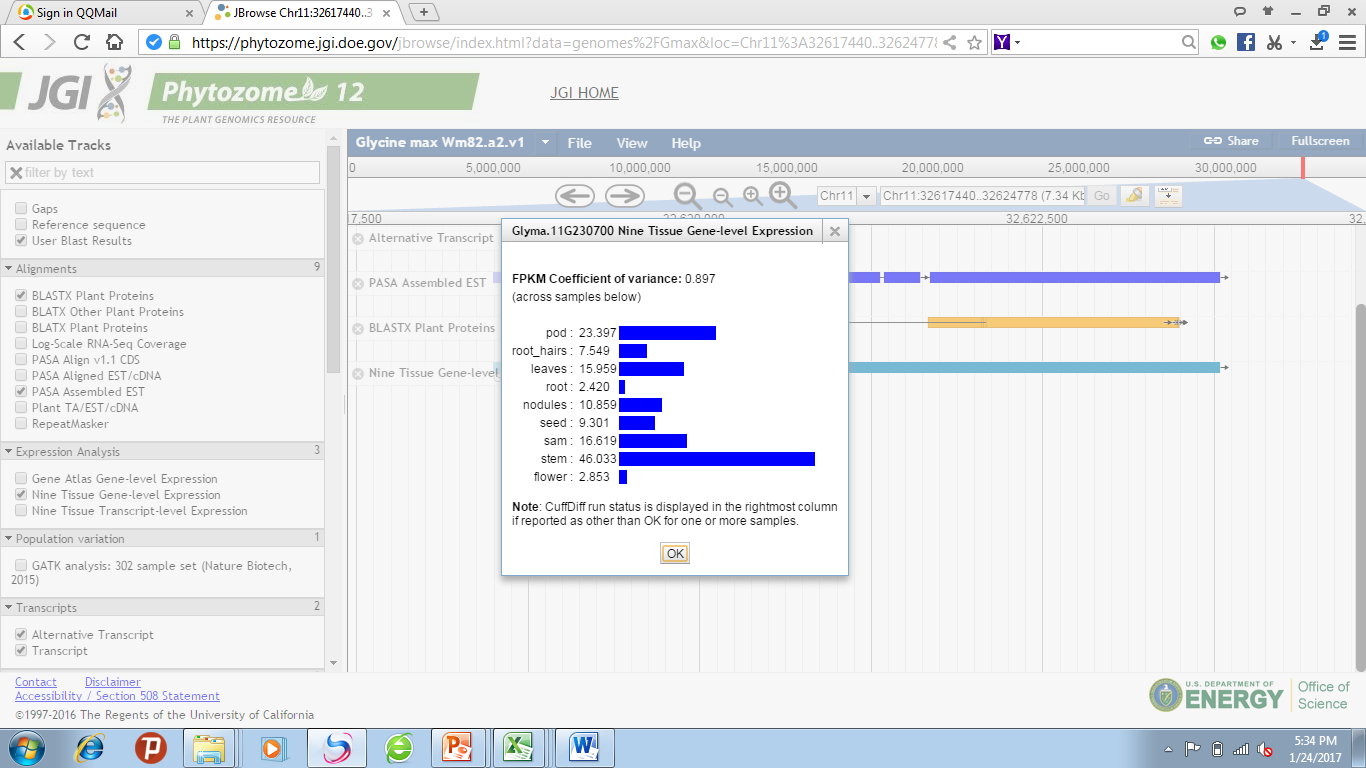

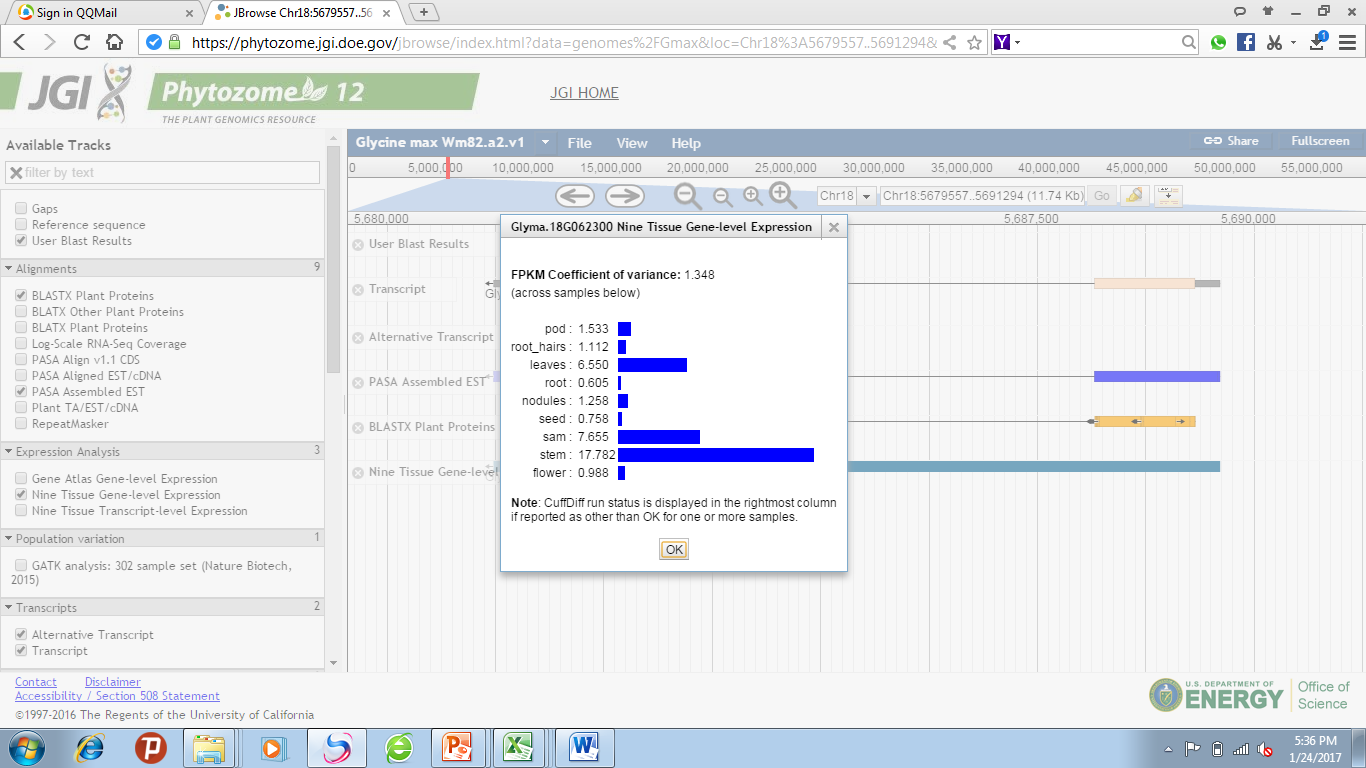


**Figure S1. Expression pattern of SL signaling genes in soybean tissues**.

Expression patterns of SL biosynthesis genes in different tissues of soybean plant. The public data are shown in phytozome (https://phytozome.jgi.doe.gov/). *GmD53a* (GLYMA11G35410), *GmD53b* (GLYMA18G06990).

**Figure S2. Expression pattern of SL signaling gene in nodule development stages of soybean**. Expression of *GmD53a* gene at different stages of nodule development and senescence.
